# Type I Hypersensitivity due to Basic Blue 99 in a Hair Colour Conditioning Agent

**DOI:** 10.1111/cod.14755

**Published:** 2025-01-13

**Authors:** Takafumi Numata, Kazuki Fujimori, Kana Kato, Tomonobu Ito, Kazutoshi Harada, Yukari Okubo

**Affiliations:** ^1^ Department of Dermatology Tokyo Medical University Tokyo Japan

**Keywords:** case report, CAS no. 68123‐13‐7, hair dye, p*‐*phenylenediamine, prick test

Basic Blue 99 (BB99) is used as a direct, nonoxidative hair colourant in hair dyes [1]. Type I hypersensitivity to BB99 has rarely been reported [2, 3, 4, 5]. We herein present a case of Type I hypersensitivity caused by BB99 and review four, previously reported cases.

## 1 Case Report

A 54‐year‐old, female, Japanese patient visited our dermatology department following a recent episode of Type I hypersensitivity. She had a history of atopic dermatitis, cold urticaria, and contact dermatitis caused by *p‐*phenylenediamine (PPD)‐containing hair dye. Moreover, 4 years previously, following the self‐application of a hair colour conditioning agent without PPD, she experienced a pruritic facial rash, which subsided overnight.

**FIGURE 1:**
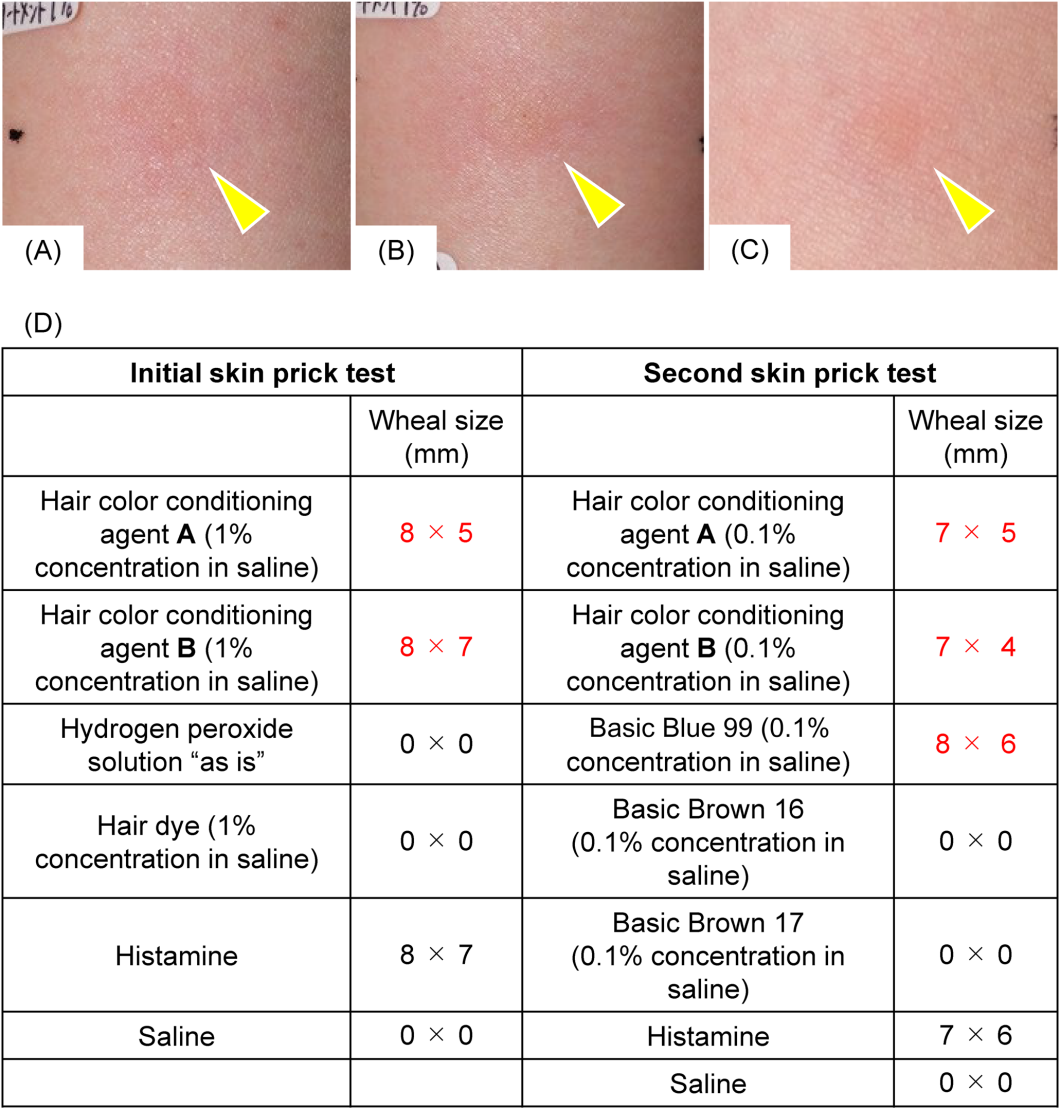
The results of the skin prick tests were positive. (A) Hair colour conditioning agent **A** at a 1% dilution in saline (arrowheads; wheal size: 8 mm × 5 mm; ++, = histamine). (B) Hair colour conditioning agent **B** at a 1% dilution in saline (arrowheads; wheal size: 8 mm × 7 mm; +++, = histamine). (C) Basic Blue 99 at a 0.1% dilution in saline (arrowhead; wheal size: 8 mm × 6 mm; +++, > histamine). (D) Summary of the skin prick test results.

One month prior to her current presentation, she experienced a pruritic facial rash, throat irritation, sneezing, stomach pain, and vomiting 20 min after a non‐PPD hair dye, a hair colour conditioning agent, and a hydrogen peroxide solution had been applied to her hair in a hair salon. She visited an emergency centre at a nearby hospital where Type I hypersensitivity reaction triggered by the hair dye or hair colour conditioning agent was diagnosed. A skin prick test carried out with the same hair colour conditioning agent which she used 4 years ago (Hair colour conditioning agent **A**) and the hair colour conditioning agent used most recently at the hair salon (Hair colour conditioning agent **B**), both at a 1% dilution in saline, as well as hair dye at a 1% dilution in saline and the hydrogen peroxide solution ‘as is’, was positive for both hair colour conditioning agents but was negative for the hair dye and hydrogen peroxide solution within 15 min after application (Figure 1A,B,D). A skin prick test later performed using the ingredients of the hair colour conditioning agents and the two hair colour conditioning agents was positive for BB99 and the two hair colour conditioning agents, but was negative for Basic Brown 16 and Basic Brown 17 within 15 min after application (Figure 1C,D). Both hair colour conditioning agents contained BB99, leading to the diagnosis of Type I hypersensitivity induced by BB99.

## 2 Discussion

BB99 (3‐[(4‐amino‐6‐bromo‐5,8‐dihydro‐1‐hydroxy‐8‐imino‐5‐oxo‐2‐naphthalenyl) amino]‐N,N,N‐trimethyl benzenaminium chloride [Arianor Steel Blue; CAS no. 68123‐13‐7]) can cause Type I hypersensitivity, including contact urticaria and contact anaphylaxis. Besides the present case, only four other cases of Type I hypersensitivity caused by BB99 have been reported to date (Table 1). Interestingly, all the patients were older than 50 years, and most were female. Two patients had a history of an atopic disorder. The positive concentration used for the skin prick tests ranged from 0.1% to 1%.

**TABLE 1:** Summary of past case reports of Basic Blue 99‐induced Type I hypersensitivity.

Author	Year	Age	Sex	Past medical history	Symptoms	Diagnosis	Positive concentration of BB99 in the SPT
Washio et al. [2]	2017	56	F	Asthma	Wheals, nausea, dyspnea, impaired consciousness	Contact anaphylaxis	0.1% aqua
Vanden Broecke et al. [3]	2014	57	F	CD to PPD, nickel, chromium, cobalt, colophonium	Itching, ‘bad taste’ in the mouth	Contact urticaria syndrome	1% aqua
Jagtman [4]	1996	71	F	ND	Severe itching, urticaria	Contact urticaria	ND
Wigger‐Alberti, Elsner, and Wüthrich [5]	1996	67	M	ND	Rhinoconjunctivitis, coughing, swelling of eyelid	Immediate‐type allergy	ND
Our case		54	F	Atopic dermatitis, cold urticaria	Pruritic facial rash, irritated throat, sneeze, stomach pain, vomiting	Type I hypersensitivity	0.1% saline

In conclusion, hair dyes containing BB99 are commonly used worldwide, but BB99 itself has rarely been reported as a contact sensitizer. The skin prick test is a useful method of identifying the allergenic substances, such as BB99. The present study found that a 0.1% dilution was sufficient for a prick test with BB99 and BB99‐containig hair colour conditioning agent.

## Author Contributions

**Takafumi Numata:** writing – original draft, methodology. **Kazuki Fujimori:** investigation. **Kana Kato:** investigation. **Tomonobu Ito:** investigation. **Kazutoshi Harada:** writing – review and editing. **Yukari Okubo:** writing – review and editing, supervision.

## Consent

Informed written consent for the publication of the details of this case and the accompanying images was obtained from the patient.

## Conflicts of Interest

The authors declare no conflicts of interest.
